# Functional dissection of the role of UHRF1 in the regulation of retinoblastoma methylome

**DOI:** 10.18632/oncotarget.17078

**Published:** 2017-04-13

**Authors:** Guangyan Kan, Heng He, Qi Zhao, Xiubo Li, Min Li, Huasheng Yang, Jong Kyong Kim

**Affiliations:** ^1^ State Key Laboratory of Ophthalmology, Zhongshan Ophthalmic Center, Sun Yat-Sen University, Guangzhou 510060, China; ^2^ Department of Immunology, Zhongshan School of Medicine, Sun Yat-Sen University, Guangzhou 510060, China; ^3^ Sun Yat-Sen University Cancer Center, State Key Laboratory of Oncology in South China, Collaborative Innovation Center for Cancer Medicine, Guangzhou 510060, China

**Keywords:** retinoblastoma, DNA methylation, UHRF1, global hypomethylation, tumorigenesis of murine retinoblastoma

## Abstract

UHRF1 (ubiquitin-like with PHD and RING finger domains 1) is a critical regulator for DNA methylation, and its frequent overexpression in human cancers has been associated with tumor-promoting effects. However, whether the overexpressed UHRF1 contributes to the establishment and maintenance of tumor methylomes and whether this process can affect the tumorigenesis remain unclear. In this study, we show that UHRF1 is highly expressed in retinoblastoma, and genomes of human primary retinoblastoma and cell lines have differential DNA methylation patterns compared with those of normal retina, characterized by lower global methylation and higher promoter methylation of tumor suppressors. However, our genome-wide DNA methylation study uncovers that UHRF1 down-modulation in retinoblastoma cells exerts minor effects on the existing methylation patterns at both bulk genome and individual gene loci, suggesting that retinoblastoma methylome is primarily maintained by other mechanisms. Furthermore, using two murine retinoblastoma models, we found that high UHRF1 expression does not alter global methylation levels in both premalignant neonatal retina and retinoblastoma tumors, implying that DNA hypomethylation may not be an early mechanism driving retinoblastoma tumorigenesis unlike what has been proposed for other types of cancer. These results suggest that tumor-promoting functions of UHRF1 in retinoblastoma are largely independent of its role in DNA methylation.

## INTRODUCTION

Ubiquitin-like with PHD and RING finger domains 1 (UHRF1) is a multi-domain epigenetic factor that has a major role in maintaining DNA methylation through cell divisions by recruiting DNA methyltransferase 1 (DNMT1) during replication [1–3]. Subsequent studies discovered that binding of histone methylation marks and ubiquitination of histone H3 by UHRF1 also contribute to the maintenance methylation, highlighting unique functions of UHRF1 coupling DNA methylation and histone modifications [4, 5]. In addition to maintenance methylation, UHRF1 plays an important role in heterochromatin formation and regulation of gene expression at individual loci [6–8]. These functions are attributed to the presence of distinct domains in UHRF1, which allows the protein to bind to histones and their modifications and also to recruit chromatin modifiers such as DNMT1, histone deacetylase 1 (HDAC1), and G9a methyltransferase [9–11].

The expression of UHRF1 in non-cancer cells is regulated along the cell cycle which culminates at late G1 phase and is required for the S phase entry in some cell types [12, 13]. Consistent with this observation, UHRF1 is expressed in proliferative cells and tissues whereas highly differentiated tissues do not express UHRF1 [8]. In cancer cells, UHRF1 is frequently found to be overexpressed constitutively, and the molecular mechanisms underlying UHRF1 overexpression are largely unknown except for a few cases where UHRF1 expression is driven by deregulated miRNAs [14–16].

As an epigenetic regulator, UHRF1 is known to contribute to tumor development by introducing changes in DNA and histone methylation by recruiting chromatin modifiers and thereby altering the gene expression. The best characterized examples observed in cancer cells are promoter hypermethylation of tumor suppressors exemplified by p16^INK4A^, BRCA1 and PPARγ [17]. Recently, transgenic overexpression of UHRF1 in normal zebrafish hepatocytes was shown to induce DNA hypomethylation, and vector-mediated UHRF1 overexpression in esophageal squamous cell carcinoma (ESCC) cell lines was reported to cause global hypomethylation [18, 19]. These studies suggest that UHRF1 overexpression may be a mechanism underlying global DNA hypomethylation in human cancers, implying a potential contribution of UHRF1 to establishment of cancer methylomes.

Retinoblastoma is an intraocular tumor which arises from developing retina by *RB1* gene inactivation [20]. A recent study showed that UHRF1 is highly expressed in a subset of human retinoblastoma and down-regulation of UHRF1 significantly reduces the size of retinoblastoma tumors grown in orthotopic xenograft models [21]. Unlike most human cancers where the mechanisms driving the UHRF1 overexpression are unclear, retinoblastoma was proposed to have a clear genetic alteration that can be connected with high expression of UHRF1 [21]. Biallelic inactivation of *RB1* gene unleashes E2F activities which in turn transcriptionally induce the expression of UHRF1. Indeed, several studies identified UHRF1 as a direct target of E2F1 in various cell systems [10, 22], and genetic disruption of *E2f1* or *E2f3* in a murine retinoblastoma model was shown to ablate UHRF1 expression in *Rb1*-knockout retinae that would otherwise exhibit high UHRF1 expression [21]. These experimental evidences demonstrate that *Rb1* inactivation may be implicated in the high expression of UHRF1 in retinoblastoma through deregulated E2F proteins. Given the well-documented roles of UHRF1 in DNA methylation, high expression of UHRF1 in retinoblastoma was hypothesized to have a critical impact on the arrangement of retinoblastoma methylome and tumorigenesis processes. However, there have been no reported studies in which the hypothesis is experimentally evaluated.

In this study, we investigated the functions of UHRF1 in the regulation of DNA methylation in retinoblastoma and assessed the contribution of global DNA methylation changes to retinoblastoma tumorigenesis.

## RESULTS

### High expression of UHRF1 in human primary retinoblastoma and cell lines

**Figure 1:**
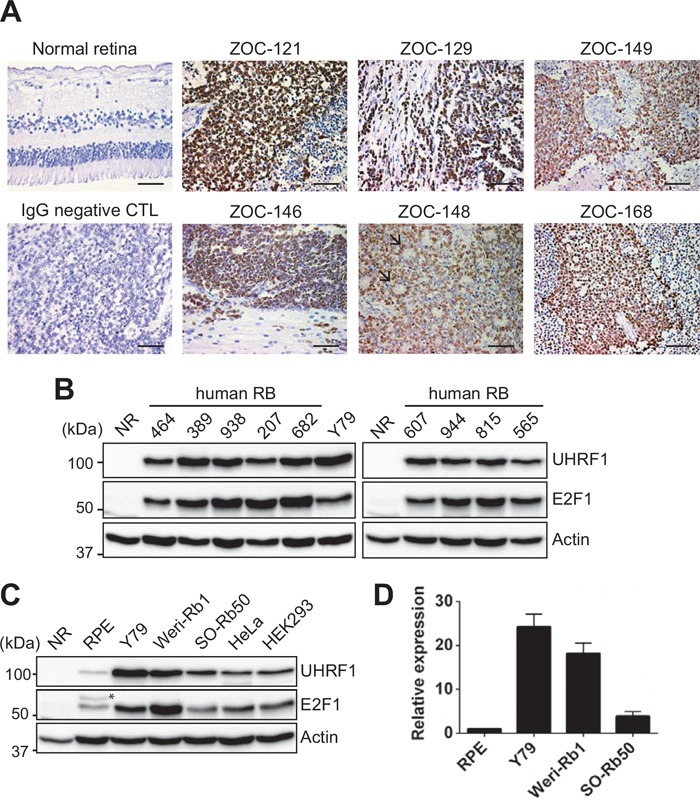
High expression of UHRF1 in primary retinoblastoma and cell lines **(A)** Immunostaining of UHRF1 in human retinoblastoma and adult normal retina (42 years of age) sections with parallel negative control (CTL) staining with mouse IgG. Nuclei were counterstained with hematoxylin. Black arrows in ZOC-148 indicate rosettes characteristic of differentiated retinoblastoma. Scale bar: 50 μm. **(B)** Expression of UHRF1 and E2F1 in human primary retinoblastoma and normal retina (NR, 12 years) determined by immunoblots. **(C)** Western blot analyses for UHRF1 and E2F1 expression in retinoblastoma and other cell lines indicated. RPE: retinal pigment epithelium. * non-specific band. **(D)** qRT-PCR analysis of relative UHRF1 expression in cells indicated. The bar graph is shown as the mean ± standard deviation (SD) of fold changes from three independent experiments, relative to the normalized UHRF1 expression level in RPE.

We examined human primary retinoblastoma and normal retina (NR) for UHRF1 expression. All of the examined tumors exhibited high UHRF1 expression while NR did not have any detectable expression of UHRF1 (Figure 1A and 1B). The high UHRF1 expression was accompanied by a high level of E2F1 expression which was also observed in retinoblastoma by others [23] (Figure 1B). This supports the prior notion that UHRF1 expression may be driven by E2F1 deregulated by the absence of functional *RB1* gene. In addition to primary tumors, we also verified the results in retinoblastoma cell lines (Y79, Weri-Rb1, and SO-Rb50), demonstrating that UHRF1 is highly expressed at both protein and transcript levels (Figure 1C and 1D).

### Differential methylation between normal retina and retinoblastoma

**Figure 2:**
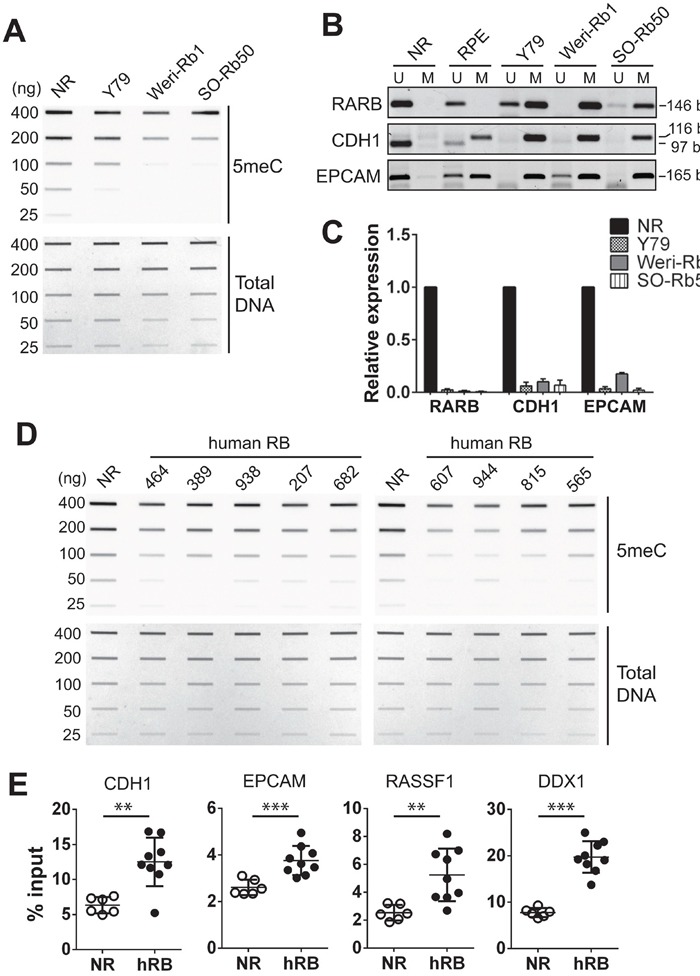
Differential methylation in retinoblastoma cells and primary tumors compared with normal retina **(A)** Total 5meC level in normal retina (NR, 12 years) and indicated cell lines determined by a slot blot analysis with serially diluted genomic DNA on the membrane stained with methylene blue as a loading control (Total DNA). **(B)** MSP for the promoters of indicated tumor suppressors. The PCR product size of unmethylated (U) and methylated (M) primers is shown on the right. **(C)** Relative expression of tumor suppressors shown in **(B)** determined by qRT-PCR. The bar graph represents the mean ± SD of fold changes from three independent experiments, relative to the normalized expression level of each tumor suppressor in NR (12 years). **(D)** Total 5meC level in NR (12 years) and human retinoblastoma determined by slot blots. **(E)** Methylation levels at the indicated gene promoters determined by MeDIP-qPCR in NR (n=6, 12-51 years of age) and human primary retinoblastoma (hRB, n=9). The graph is shown as the mean ± SD and the statistical analysis was performed by Mann-Whitney test (two-tailed). ** *P* < 0.01, *** *P* < 0.001.

We next examined the total methylation levels in genomes of NR and retinoblastoma cell lines by slot blots using a 5-methylcytosine (5meC)-specific antibody. Compared with NR, all three retinoblastoma cell lines showed a low level of global DNA methylation (Figure 2A). In addition to the total methylation, we examined the methylation status at specific gene loci where higher promoter methylation was reported in other cancers, but not in retinoblastoma yet [24–26]. In NR genome, promoters of the three tumor suppressors (*RARB*, *CDH1*, and *EPCAM*) were found to be almost exclusively unmethylated by methylation-specific PCR (MSP) (Figure 2B). In contrast, the three retinoblastoma cell lines displayed mostly methylated promoters on the identical loci. Moreover, the differential methylation status on these promoters inversely correlated with the gene expression level (Figure 2C). We also examined a subset of primary tumors for total methylation levels as compared with NR. Although the lower global DNA methylation in tumor genomes is not as distinct as in retinoblastoma cell lines, it was observed for all examined tumors. (Figure 2D). Furthermore, differential methylation was detected at selected promoters between primary tumors and NR by methylated DNA-immunoprecipitation (MeDIP) analyses (Figure 2E). The comparable total methylation level and enrichment of methylation signals at specific loci among different NR tissues indicated that the NR tissue that we commonly used for a series of methylation analyses can serve as a representative control group (Supplementary Figure 1 and Figure 2E). These data demonstrate that there are distinct differences in DNA methylation between normal retina and retinoblastoma in global and loci-specific manners.

### Effects of UHRF1 knockdown on DNA methylation in retinoblastoma cells

**Figure 3:**
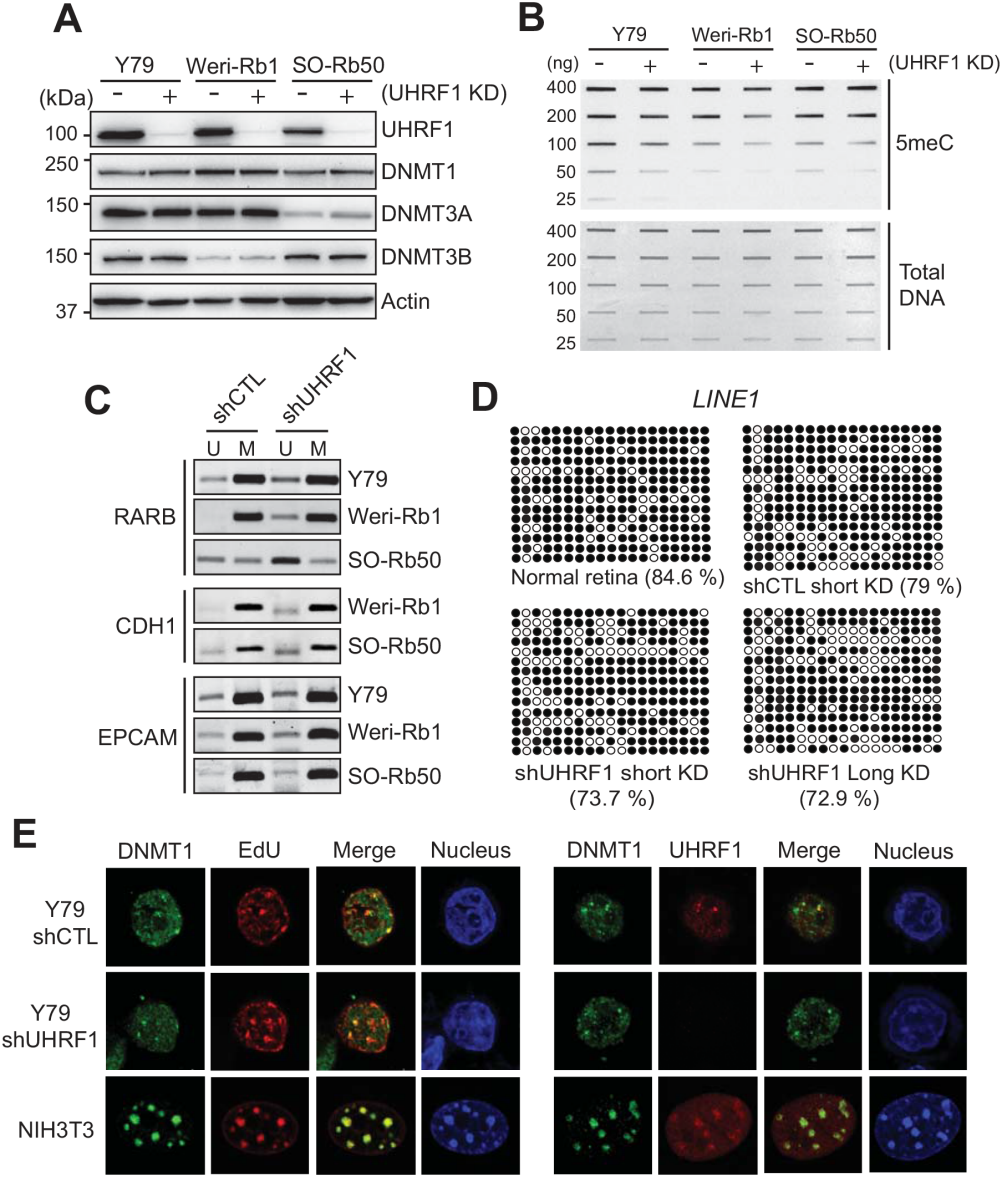
Effects of UHRF1 knockdown on DNA methylation in retinoblastoma cells **(A)** Expression of UHRF1 and indicated DNMT family proteins in retinoblastoma cell lines after lentiviral transduction of constructs harbouring shRNA against UHRF1 (+) or non-specific control shRNA (−). **(B)** Total 5meC level in retinoblastoma cells after long-term (stable) UHRF1 knockdown (KD) determined by a slot blot analysis. **(C)** MSP for the promoters of indicated tumor suppressors in retinoblastoma cells after long-term control KD (shCTL) or UHRF1 KD (shUHRF1). The PCR products of unmethylated (U) and methylated (M) primers are shown. **(D)** Bisulfite sequencing showing methylation patterns at LINE1. Y79 cells were subjected to short-term or long-term, control or UHRF1 KD as indicated, and shown with adult normal retina (42 years). Methylated and unmethylated CpG dinucleotides are represented by closed and open circles, respectively. Percentage of methylated CpG dinucleotides is shown in parentheses. **(E)** DNMT1 recruitment to replication foci (left panel) and colocalization of DNMT1 and UHRF1 during S phase (right panel). Control or UHRF1 knockdown Y79 cells were enriched for S phase by aphidicolin treatment and subsequent release to mid- to late S phase. DNA replication foci were labelled by EdU incorporation during the release to S phase and nuclei were counterstained with DAPI. DNMT1 and UHRF1 were visualized by immunostaining. Images of NIH3T3 cells at S phase are shown at the same magnification as Y79 cells as a positive control.

Since UHRF1 expression is high and differential DNA methylation exists in retinoblastoma as compared with normal retina, we next investigated the effects of UHRF1 knockdown on global and gene-specific methylation in retinoblastoma cells. Lentiviral transduction of UHRF1 shRNA efficiently reduced the UHRF1 protein level in retinoblastoma cell lines while the expression level of DNMT family proteins was not concomitantly changed (Figure 3A and Supplementary Figure 2). Using stable knockdown cells, we could detect a modest decrease in global methylation and a small degree of demethylation at specific loci upon UHRF1 down-regulation (Figure 3B and 3C). We suspected that our routine stable knockdown conditions may not be sufficient to detect DNA methylation changes during mitotic inheritance, however, the experiments using the cells selected on puromycin for 25 days also gave the similar results (data not shown). Furthermore, bisulfite sequencing on the promoter of long interspersed nuclear element 1 (LINE1) which is a retrotransposon [27] revealed that the methylation was not much reduced for both short-term and long-term UHRF1 knockdown compared with control knockdown (Figure 3D), suggesting that maintenance methylation in retinoblastoma cells is not severely affected by UHRF1 depletion. The lack of obvious effects of UHRF1 knockdown on DNA methylation maintenance in Y79 was not due to proliferation defects (Supplementary Figure 3), and reflected a biological feature of Y79 cells since the identical shRNA-mediated stable knockdown in 293T and HeLa cells resulted in substantial defects in DNA methylation maintenance (Supplementary Figure 4). To gain further understanding of this phenomenon, we examined whether DNMT1 can be normally recruited to replication foci during S phase in Y79 cells in the presence or absence of stable knockdown of UHRF1. For both control and UHRF1 knockdown cells, DNMT1 showed a diffuse pattern with a very few distinct foci formed in nuclei and only part of EdU-labelled replication foci colocalized with DNMT1 foci, which contrasts with the case of NIH3T3 shown as a positive control (Figure 3E, left panel). Similarly, UHRF1 in Y79 cells formed only a few foci and did not entirely colocalize with DNMT1 during S phase (Figure 3E, right panel). Since the canonical function of DNMT1 in maintenance methylation is preserved in Y79 cells as revealed by global hypomethylation upon 5-azacytidine treatment (Supplementary Figure 5), the results imply that fine-tuned regulation of DNMT1 function during S phase may be partially impaired in retinoblastoma cells, rendering the subnuclear localization pattern of DNMT1 and maintenance methylation less sensitive to UHRF1 status. Despite the deregulated DNMT1 recruitment during replication, DNA methylation maintenance was not severely affected in Y79 cells, which may suggest that other DNMT family proteins might also be involved in maintenance methylation in retinoblastoma cells although DNMT1 plays a dominant role. In support of this possibility, DNMT3A and DNMT3B were found to be highly expressed in Y79 and primary retinoblastoma (Supplementary Figure 6).

### Genome-wide analysis of differentially methylated regions

We also performed MeDIP-sequencing to uncover any potential effects of UHRF1 knockdown on DNA methylation in retinoblastoma by genome-wide examination of relative methylation differences between control and UHRF1 knockdown Y79 cells. Prior to the genome-wide analysis of differentially methylated regions (DMRs) in Y79 cells upon UHRF1 knockdown, we generated and sequenced MeDIP libraries from NR and Y79 cells to examine the differences in Y79 methylation patterns compared with those of NR. The evaluation of relative CpG enrichment over the reference genome revealed that the immunoprecipitation with a 5meC antibody was efficient (Supplementary Table 1), and the saturation analysis showed that the sequencing depth that we employed provided sufficient coverage over CpG sites in the genome (Supplementary Figure 7).

**Figure 4:**
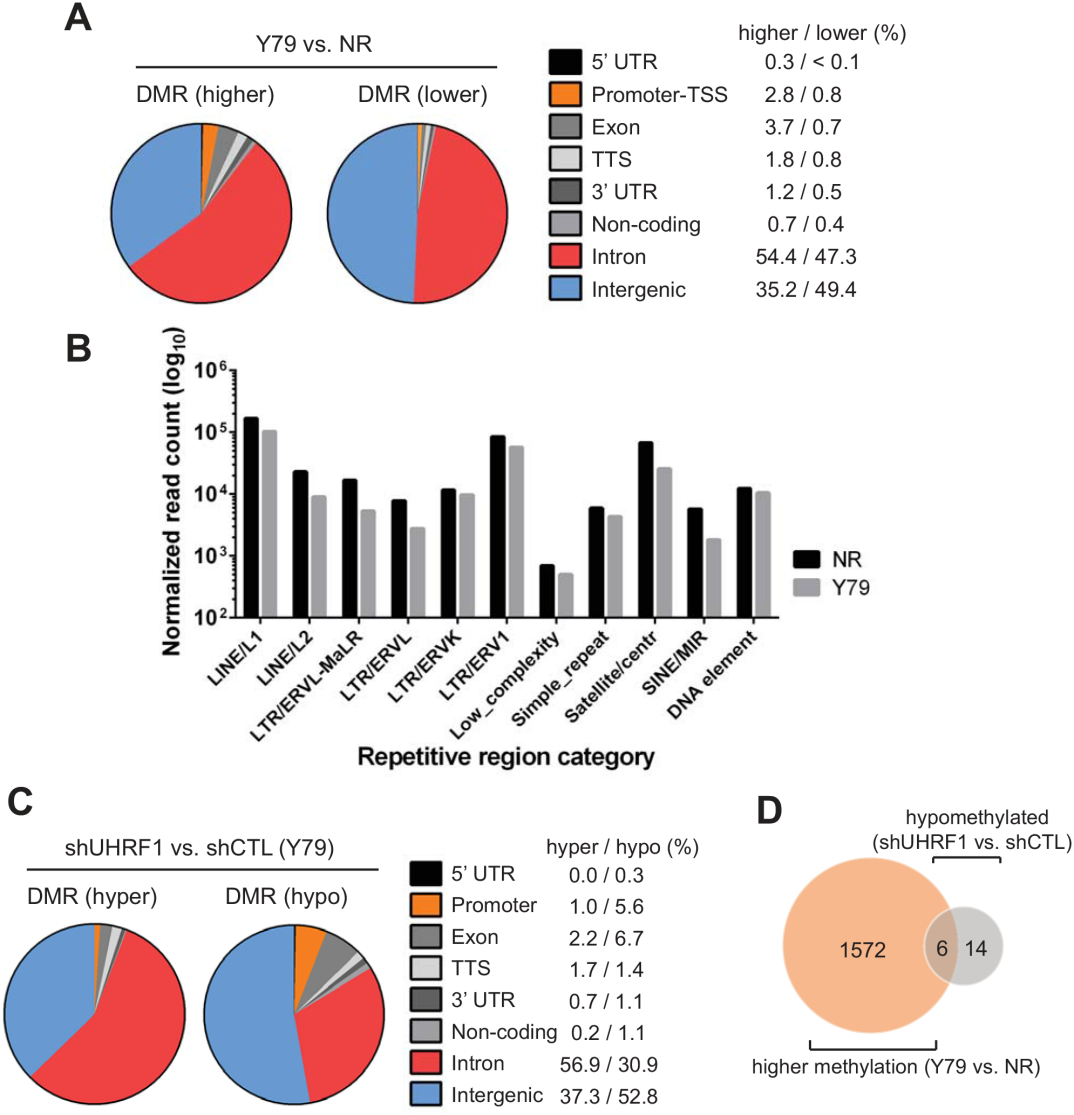
Genome-wide analyses of differentially methylated regions **(A)** Distribution of differentially methylated regions (DMRs) in Y79 retinoblastoma cells as compared with normal retina (NR, 12 years). DMRs are divided into higher methylation (higher) and lower methylation (lower) regions in Y79, and their relative proportion on different genomic regions is shown as a pie chart with percentages of DMRs located at each category of genomic regions on the right. **(B)** Relative methylation differences in repetitive elements between NR and Y79. Sequence reads that are mapped to different classes of repetitive regions were normalized by the number of total mapped reads/10^6^ of the corresponding libraries and represented in log_10_ scale on Y axis. **(C)** Distribution of DMRs in UHRF1 knockdown Y79 cells in comparison with control knockdown cells. Pie charts and the percentages are shown as in **(A)**. **(D)** Venn diagram showing the overlap between the promoters with higher methylation in Y79 than in NR and hypomethylated promoters in UHRF1 knockdown Y79 cells in comparison with control knockdown.

When we determined the DMRs in Y79 cells in comparison with NR, 77382 total DMRs consisting of 57265 regions with higher methylation and 20117 regions with lower methylation could be identified (Supplementary Table 2 and Figure 4A). Most of DMRs were found to be located in introns and intergenic regions for both categories of DMRs, however, a substantially larger proportion of the DMRs with higher methylation in Y79 were identified to be promoters and exons than was the case of DMRs with lower methylation (Figure 4A). Considering the lower global methylation in Y79 cells than that of NR (Figure 2A), the larger proportion and number of non-intergenic DMRs with higher methylation in Y79 may suggest that Y79 methylome takes on distinct methylation patterns while methylation marks in NR are distributed more evenly across the bulk genome. Consistent with this notion, repetitive element analyses revealed that NR has a higher level of methylation in major classes of repetitive elements than Y79 (Figure 4B). For identification of DMRs between the control and UHRF1 knockdown Y79 cells, we analysed three independent sets of sequencing data to unambiguously determine the effects of UHRF1 knockdown on the methylome of Y79 cells. Applying the same analysis criteria used for the comparison of Y79 versus NR to the DMR analysis for control and UHRF1 knockdown Y79 cells resulted in extremely few targets. To ensure that we do not miss any potentially important DMRs, we employed the less stringent filtering criteria and could detect 769 DMRs consisting of 413 hypermethylated and 356 hypomethylated regions in UHRF1 knockdown cells compared with their control knockdown counterpart (Supplementary Table 3 and Figure 4C). Of note, a higher proportion of hypomethylated regions were found to be promoters and exons than was the case of hypermethylated DMRs (Figure 4C). This may suggest that promoters with high methylation in Y79 cells as compared with NR might have undergone demethylation upon UHRF1 knockdown. Among the 1578 promoters with higher methylation in Y79 than in NR (Supplementary Table 2), only 6 promoters were found to undergo demethylation in UHRF1 knockdown cells (Figure 4D), indicating that most promoters with higher methylation in Y79 cells are not affected by UHRF1 knockdown. Consistent with the results, pairwise Pearson's correlations among the three independent sets of knockdown libraries showed high correlation coefficients of > 0.91 for all comparisons (Supplementary Table 4, shaded in grey). This demonstrates that UHRF1 knockdown cells have highly similar methylation patterns to those of control knockdown cells, resulting in few DMRs detected from the comparisons.

### Differentially methylated promoters in Y79 compared with NR are largely unaffected by UHRF1 depletion

**Figure 5:**
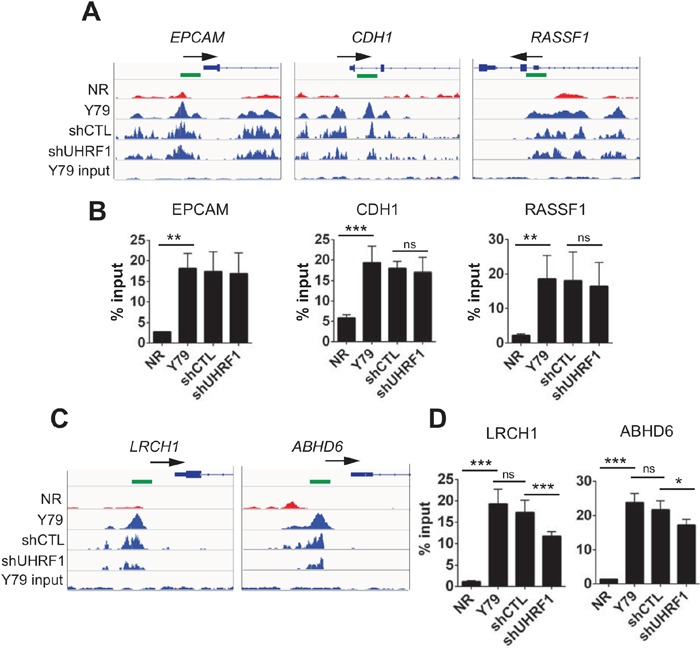
Effects of UHRF1 knockdown on promoter methylation of selected gene loci **(A, C)** IGV browser tracks showing the distribution of 5meC signals on the indicated promoter regions in the five libraries as shown on the left. The NR indicates normal retina (12 years) and the identical retina tissue was used for the subsequent validation experiments. The shCTL and shUHRF1 represent the control and UHRF1 knockdown Y79 libraries, respectively. Maximal height of the tracks was set identically within the groups. Arrows mark the direction of transcription on the gene structure shown and green squares indicate the location of primers used for MeDIP-qPCR. **(B, D)** Validation of 5meC enrichment on the promoter regions shown in **(A)** and **(C)** by MeDIP-qPCR. The bar graphs are represented as mean ± SD of % input, and the statistical analysis was performed by unpaired student's t-test (two-tailed). ** *P* < 0.01, *** *P* < 0.001, ns: not significant.

To validate our genome-wide DMR analysis results, we performed MeDIP-qPCR on a few selected promoters using independently prepared MeDIP samples (Figure 5). Promoters of the three tumor suppressors (*EPCAM*, *CDH1*, *RASSF1*) indeed had higher methylation in Y79 than in NR, and the methylation of these promoters was unaffected by the UHRF1 knockdown (Figure 5A and 5B). We also examined a few DMR promoters detected from the comparison between control and UHRF1 knockdown Y79 cells. Although the differences were modest, statistically significant reduction in methylation signal was detected upon UHRF1 knockdown (Figure 5C and 5D), but the modest reduction in promoter methylation did not affect the gene expression levels (data not shown).

Taken together, our genome-wide methylation analysis in Y79 retinoblastoma cells revealed that UHRF1 does not exert much effect on the overall methylation patterns, unlike what has been predicted based on the studies in other cancer cells.

### High UHRF1 expression does not alter global methylation in murine retinoblastoma models

**Figure 6:**
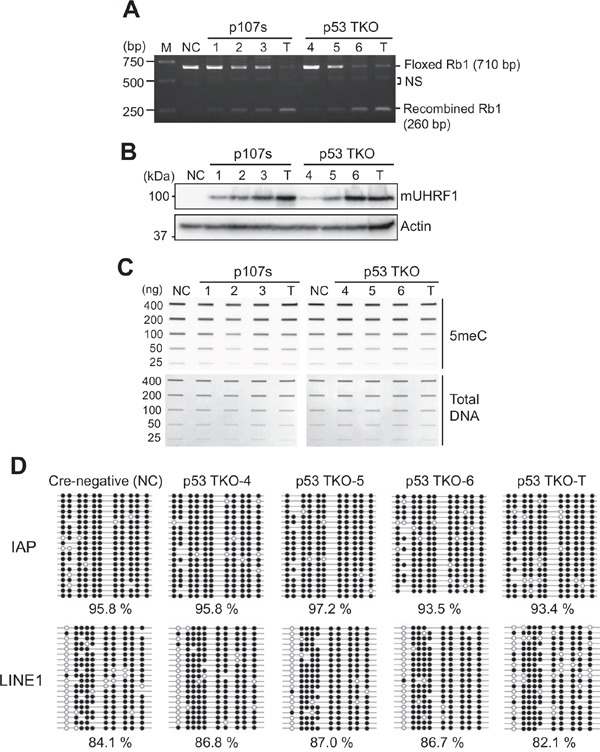
High UHRF1 expression does not change global DNA methylation in murine retinoblastoma models **(A)** Genomic DNA PCR showing a different level of recombination in *Rb1* allele by Chx10-Cre activity in the retina of two mouse retinoblastoma models (p107s and p53 TKO). P8 retina (1-3) and tumor (T) tissues of p107s (*Chx10-Cre, Rb1^lox/lox^, Rbl2^−/−^, Rbl1^+/−^*) were analysed in parallel with P8 retinae (4-6) and tumor (T) from p53 TKO (*Chx10-Cre, Rb1^lox/lox^, Tp53^lox/lox^, Rbl1^−/−^*). NC (negative control) indicates Cre-negative control retina of the p53 TKO line. The PCR product size of *Rb1* allele flanked by *loxP* (Floxed) and recombined *Rb1* is shown on the right. NS indicates non-specific bands. **(B)** Expression of mouse UHRF1 (mUHRF1) in the corresponding tissues shown in **(A)**. **(C)** Slot blot analyses for the total 5meC level in the corresponding samples described in **(A)**. **(D)** Bisulfite sequencing that shows methylation patterns at IAP and LINE1 for the p53 TKO P8 retinae (4-6) and tumor (T) shown in **(C)**. Methylated and unmethylated CpG dinucleotides are represented by closed and open circles, respectively. Percentage of methylated CpG dinucleotides is shown below.

A recent study reported that transgenic overexpression of UHRF1 in zebrafish hepatocytes can drive hepatocellular carcinoma by inducing global DNA hypomethylation and subsequently bypassing senescence [18]. The study proposed that global hypomethylation caused by UHRF1 overexpression may be an initiating mechanism underlying neoplastic transformation of normal hepatocytes to HCC. As in human retinoblastoma, UHRF1 is highly expressed in mouse retinoblastoma which is known to recapitulate many characteristics of human retinoblastoma [21, 28]. Therefore, we investigated if high expression of UHRF1 can decrease global DNA methylation levels in mouse retinoblastoma models. We employed eye-specific conditional knockout models where *Rb1* and *Tp53* alleles are recombined by Chx10-Cre transgene. Recombination mediated by Chx10-Cre occurs as early as E10.5 in retinal progenitor cells in a mosaic pattern [29]. Due to the mosaicism of Chx10-Cre expression, recombination efficiency of conditional alleles varies in the mouse models, which allowed us to screen retinae with a different level of *Rb1* recombination. By taking genomic DNA from postnatal day 8 (P8) retina of p107s and p53 TKO mice, we screened three pups with low to high recombined *Rb1* from each mouse model (Figure 6A). As a control, *Rb1* recombination in tumors was examined in parallel, and found to be highly efficient as expected (Figure 6A). Then, we examined the UHRF1 expression in the corresponding tissues with different levels of *Rb1* recombination (Figure 6B). The UHRF1 protein level positively correlated with *Rb1* inactivation level from no detectable expression in Cre-negative retina to high expression in tumors. Next, total methylation levels were measured in the corresponding genomic DNA to check whether the high UHRF1 expression can decrease global DNA methylation as reported in the transgenic zebrafish model [18]. Unlike the previous study, we did not see any discernable decrease in global methylation in retinae expressing a high level of UHRF1 and tumors as compared with retinae expressing a low level of UHRF1 (Figure 6C). To further verify this result, we performed bisulfite sequencing on intracisternal A particle (IAP) and LINE1 as surrogate indicators for global methylation in the same p53 TKO P8 and tumor tissues. Consistent with the slot blot result (Figure 6C), there was no clear correlation between UHRF1 expression level and the global methylation (Figure 6D). Furthermore, similar results were obtained when we performed the bisulfite sequencing on the p107s P8 and tumor tissues (Supplementary Figure 8). These results indicated that high UHRF1 expression does not induce changes in global methylation in retinae and possibly in retinal tumors as well, and led us to further investigate the contribution of global DNA methylation to retinoblastoma tumorigenesis.

### Global hypomethylation may not be a critical early mechanism driving retinoblastoma tumorigenesis

**Figure 7:**
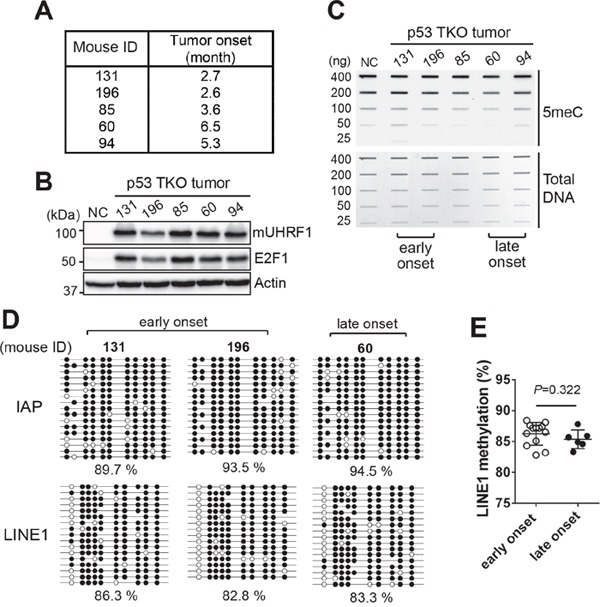
Global DNA hypomethylation may not be an early event leading to retinoblastoma development **(A)** Table showing a representative set of p53 TKO mice with a different tumor onset time. **(B)** Expression of mouse UHRF1 (mUHRF1) and E2F1 in the tumors described in **(A)**. NC indicates Cre-negative control retina of the p53 TKO line. **(C)** Slot blot analysis for the total 5meC level in the corresponding tumors described in **(A)**. Early and late onset tumors are indicated at the bottom. **(D)** Bisulfite sequencing of IAP and LINE1 using the indicated mouse tumor DNA. Percentage of methylated CpG dinucleotides represented by closed circles is shown. **(E)** LINE1 methylation level in early onset (n=13) and late onset (n=6) p53 TKO tumors, determined by bisulfite sequencing. The graph is shown as the mean ± SD and the statistical analysis was performed by Mann-Whitney test (two-tailed).

We hypothesized that global methylation level would be significantly different between early onset and late onset tumors if global hypomethylation is a critical early mechanism driving retinoblastoma development. To test the hypothesis, we took a subset of p53 TKO mice with a different tumor onset time based on our routine inspection. These mice develop detectable tumors characterized by protruding eyeballs and a white lesion on the eyes (Supplementary Figure 9A). In our cohort of p53 TKO mice, early onset is defined as 2-3 months while late onset tumors take 5-6 months of time before detectable tumors develop (data not shown). We examined 13 mice for early onset and 7 mice for late onset tumors (Figure 7A and Supplementary Figure 9B), and the corresponding tissues were analysed for UHRF1 expression and global methylation level. The UHRF1 was highly expressed in all of mouse tumors with concomitant expression of E2F1 (Figure 7B and Supplementary Figure 9C), however, there were no consistent global methylation changes between early onset and late onset tumors (Figure 7C and Supplementary Figure 9D). Bisulfite sequencing on IAP and LINE1 using the same tumor DNA further validated the results showing no correlation between global methylation levels and tumor onset time (Figure 7D and 7E). Taken together with the P8 retina analysis (Figure 6), the data imply that global hypomethylation driven by high UHRF1 expression is unlikely to be an early event driving retinoblastoma tumorigenesis since there are no significant changes in global methylation among the retinae with different levels of UHRF1 expression and among the retinoblastoma tumors with a different onset time. Therefore, high UHRF1 expression may promote retinoblastoma development primarily through its other functions, rather than deregulation of DNA methylation.

## DISCUSSION

An increasing body of evidence suggests that UHRF1 is an oncogene, demonstrated by its tumor-promoting functions in proliferation, invasion, and regulation of apoptosis in various cancer cells [8]. These tumor-promoting functions of UHRF1 often involve its control of DNA methylation at tumor suppressors and other related genes in diverse cellular pathways. In the context of tumorigenesis, a recent study reported that transgenic UHRF1 overexpression in normal zebrafish hepatocytes induces DNA hypomethylation, and a subset of the transgenic fish develop hepatocellular carcinoma [18]. The study presents a model that UHRF1-driven DNA hypomethylation may be a potential mechanism by which normal cells undergo neoplastic transformation. In human cancers, UHRF1 is frequently overexpressed and cancer genomes often exhibit a diverse level of global DNA hypomethylation as compared with their normal tissues. Therefore, it is plausible to hypothesize that UHRF1 overexpression may directly cause DNA hypomethylation in cancer genomes, however, it is unclear in most human cancers when and how UHRF1 begins to be overexpressed over the entire course of tumorigenesis, and whether the overexpressed UHRF1 indeed reprograms cancer methylomes. In this study, we used murine retinoblastoma models where we can monitor different levels of UHRF1 expression according to the extent of *Rb1* inactivation which may be a physiological cause for high UHRF1 expression in retinoblastoma. Because the recombination by Chx10-Cre transgene occurs in retinal progenitor cells during early retinal development [29], we hypothesized that neonatal mice would have a window of opportunity to reprogram their methylome if *Rb1* recombination is efficient and thereby a high level of UHRF1 expression is achieved. Analyses of retinae from P8 mice demonstrated that high levels of UHRF1 expression can be detected in the young premalignant mice having high *Rb1* recombination. However, P8 retinae with high UHRF1 expression and tumors did not display any discernable changes in global DNA methylation compared to P8 retinae with low UHRF1 expression. Furthermore, there was no correlation between global methylation levels and tumor onset time in mouse tumors. The murine retinoblastoma models have other genetic alterations in addition to *Rb1* inactivation, however, these genetic modifications do not cause any distinguishable DNA methylation changes compared with wild-type retinae (data not shown). Therefore, our results suggest a possibility that high UHRF1 expression may not decrease global DNA methylation in mouse retinoblastoma and global DNA hypomethylation may not be an early mechanism driving retinoblastoma development, although this possibility needs to be further validated because the specific cell type from which retinoblastoma arises could not be analysed in parallel as the normal cell counterpart in this study. However, these results support prior reports that genome-wide DNA hypomethylation observed in many cancers may be a consequence of tumorigenesis rather than the cause of tumor development, as some cancers develop without apparent hypomethylation and most epigenetic studies are performed on late-stage malignant tumors which usually provide limited information on the cause and effect relationship of the tumor development [30, 31]. Our results imply that retinoblastoma may be one of the cases where DNA hypomethylation is not required for the initiation of tumor development but may occur or become detectable later by clonal selection/expansion during the progression of tumor since human primary retinoblastoma and cell lines have appreciable levels of lower global methylation than normal retina. This possibility awaits further validation due to the lack of the parallel analysis of the true normal cell counterpart for retinoblastoma and current difficulties in obtaining clear experimental evidences for the correlation between DNA methylation changes and tumor progression in human retinoblastoma where serial analyses of the same tumors along the progression are not so feasible.

Although UHRF1 has modest effects on DNA methylation in retinoblastoma cells and mouse tumors, there are distinct features in human retinoblastoma methylomes both globally and in loci-specific manners. Compared with normal retina, retinoblastoma cells displayed low global methylation and high promoter methylation of tumor suppressors, which is frequently observed in many human cancers. This suggests that other nuclear factors rather than UHRF1 may play a key role in reprograming methylomes in retinoblastoma. In this regard, high expression of three DNMT family proteins in human primary retinoblastoma and cell lines (Supplementary Figure 6) may account for the differences in the regulation of retinoblastoma methylomes, reducing the dependency on UHRF1 for the regulation of DNA methylation. Indeed, DNMT3A and DNMT3B are known to be involved in maintenance methylation in addition to their canonical functions in *de novo* methylation although DNMT1 plays a dominant role in DNA methylation maintenance [32]. In Y79 cells, fine-tuned regulation of DNMT1 functions during replication was found to be at least partially impaired while the function of DNMT1 as a maintenance methyltransferase is preserved. Therefore, the modest effects of UHRF1 depletion on DNA methylation maintenance in Y79 may suggest that other DNMT family proteins may compensate for the compromised regulation of DNMT1, independently of UHRF1. Nevertheless, UHRF1 was shown to have tumor-promoting functions in retinoblastoma development as demonstrated by impaired colony formation and reduced size of xenografted tumors upon UHRF1 knockdown in retinoblastoma cells [21]. Another related study from our laboratory also indicates that UHRF1 down-regulation is beneficial to augment the therapeutic efficacy of retinoblastoma treatment (unpublished data). Therefore, tumor-promoting effects of UHRF1 in retinoblastoma are likely to involve other functions of UHRF1 rather than its role in DNA methylation. A potential concern for targeting epigenetic factors for cancer therapy is indiscriminative impairment of their normal epigenetic activities which are essential for tissue homeostasis. Considering that normal retina does not express UHRF1, our findings suggest that local UHRF1 targeting in affected lesions may be promising for a novel retinoblastoma therapy without a risk of systemic complications in DNA methylation.

In summary, we investigated the role of UHRF1 in establishment and maintenance of DNA methylome in retinoblastoma, using mouse models and human retinoblastoma cells. Unlike what has been predicted based on its well-known functions in DNA methylation, our comprehensive methylation analyses revealed that the high level of UHRF1 expression in retinoblastoma has little effect on the establishment and maintenance of retinoblastoma methylome. This suggests that tumor-promoting functions of UHRF1 in retinoblastoma are largely independent of its role in DNA methylation, and the potential role for UHRF1 in tumor methylome regulation may be tumor type-specific. Our study on the functions of UHRF1 in DNA methylome regulation and contribution of global methylation changes to retinoblastoma tumorigenesis provides a new insight into retinoblastoma biology.

## MATERIALS AND METHODS

### Cell culture, human tissues, and murine retinoblastoma models

Y79, Weri-Rb1, and RPE cells were obtained from American Type Culture Collection (ATCC), and SO-Rb50 was established at the Zhongshan Ophthalmic Center (ZOC) [33]. All retinoblastoma cell lines were maintained in RPMI-1640 containing 10% FBS and penicillin-streptomycin (Gibco). Normal retina tissues were obtained from the ZOC eye bank. The age of normal retina donors ranges from 12 years to various adulthood ages due to difficulties in obtaining fetal retina. Most experiments in this study were performed using the 12 year-old retina tissue. Fresh human retinoblastoma tissues were removed from enucleated eye globes immediately after surgery according to the procedure described previously [34]. The study with human clinical samples was approved by the ZOC institutional review board. All human specimens used for this study were de-identified, and informed consent forms were obtained. The mouse retinoblastoma models (p107s and p53 TKO) were previously described [28] and obtained from the St. Jude Children's Research Hospital through the Childhood Solid Tumor Network (CSTN) [35]. The genotypes of the p107s and p53 TKO lines are as follows: p107s (*Chx10-Cre, Rb1^lox/lox^, Rbl2^−/−^, Rbl1^+/−^*) and p53 TKO (*Chx10-Cre, Rb1^lox/lox^, Tp53^lox/lox^, Rbl1^−/−^*). All animal studies were conducted with the approval of the Sun Yat-sen University Institutional Animal Care and Use Committee.

### Generation of stable knockdown cells

Lentiviral pLKO.1 constructs expressing shRNA specific for UHRF1 (shUHRF1) and control shRNA (shCTL) were purchased from Thermo Scientific. The lentiviral particles were produced by following the pLKO.1 packaging protocol from Addgene. Lentiviral transduction was performed after plating 5 × 10^6^ suspension retinoblastoma cells on poly-D-lysine (PDL) (0.1 mg/ml, Sigma)-coated dishes. Short-term and long-term knockdown retinoblastoma cell lines were generated by 12-hour infection followed by 4 days of incubation (short-term knockdown), and 8-9 days of selection on puromycin (1 μg/ml) in addition to the initial 4 days of incubation (long-term knockdown), respectively. When necessary, the puromycin selection was extended up to 25 days.

### Western blot

Cleared lysates (25-30 μg) were subjected to 10% SDS-PAGE, and antibodies for western blots are as follows: anti-human UHRF1 (612264, BD Biosciences), anti-mouse UHRF1 (sc-373750, Santa Cruz), anti-E2F1 (sc-193, Santa Cruz), anti-DNMT1 (5032, Cell Signalling Technology), anti-DNMT3A (sc-20703, Santa Cruz), anti-DNMT3B (NB100-56514, Novus), and anti-actin (A1978, Sigma).

### Quantitative RT-PCR

The qRT-PCR was performed by analysing samples in triplicate using at least three independent sets of cDNA. The results were normalized by the expression level of actin as an internal control. The primer sequences used for the qRT-PCR are listed in supplementary data.

### Immunofluorescence

Y79 shCTL and shUHRF1 cells were treated with aphidicolin (3 μg/ml) for 24 hr to arrest the cells at early S phase, and then released into regular media for 6 hr with 5 μM 5-ethynyl-2′-deoxyuridine (EdU) for the last 1 hr during the release. The S phase-enriched cells were plated on the PDL-coated cover glasses and subjected to immunostaining with anti-DNMT1 antibody (sc-20701, Santa Cruz) alone or together with anti-UHRF1 antibody (sc-373750, Santa Cruz). The EdU detection was carried out by following the instructions of Click-iT EdU imaging kit (Invitrogen). For enrichment of NIH3T3 cells at S phase, cells were subjected to serum starvation (0.1% FBS) for 30 hr and then re-stimulated with 10% serum for 19 hr with the last 1 hr of EdU labelling. Cell nuclei were counterstained with 4′, 6-diamidino-2-phenylindole (DAPI), and cells were visualized with a Zeiss LSM 800 confocal microscope with a 63x oil objective lens.

### Slot blot

Serially diluted genomic DNA was denatured and loaded onto nitrocellulose membrane using a slot blot apparatus (Biorad). After washing with 4X saline sodium citrate, the membrane was subjected to UV crosslinking and probed with a 5-methylcytosine antibody (C15200081, Diagenode). After chemiluminescence detection, the membrane was stained with 0.2% methylene blue in 0.3M sodium acetate (pH 5.2) as a loading control for the slot blot.

### MSP and bisulfite sequencing

Bisulfite conversion of genomic DNA (2 μg) was performed by using EpiTect Bisulfite kit (Qiagen). The bisulfite DNA was used for either MSP or amplification of indicated fragments for bisulfite sequencing following the procedure described previously [36]. The primers used for MSP and bisulfite sequencing are listed in supplementary data.

### MeDIP-sequencing and MeDIP-qPCR

MeDIP-seq libraries were prepared by following the procedure described previously [37]. MeDIP libraries from normal retina and Y79 cells were subjected to 125 bp paired-end sequencing (PE125) on an Illumina HiSeq 2500 platform while the subsequent MeDIP libraries generated from Y79 shCTL and shUHRF1 cells in triplicate were sequenced as 50 bp single-end reads (SE50). To validate the results of MeDIP-seq data, differentially methylated targets were examined for the enrichment of methylation marks by MeDIP-qPCR, using at least three independent sets of MeDIP samples. The primers used for MeDIP-qPCR are listed in supplementary data.

### Bioinformatic analyses

The fastq files were aligned to the hg19 human reference genome using Bowtie 2 [38]. By assigning the resultant uniquely mapped reads into 200 bp non-overlapping widows across the genome, peaks were identified by using SICER [39] with either input DNA or IgG-immunoprecipitated DNA as a control and with a false discovery rate (FDR) threshold of 0.05. The BAM files of the uniquely mapped reads were generated to visualize the distribution of the peaks with IGV genome browser. Relative CpG enrichment over the reference genome, the depth of coverage, and genome-wide Pearson correlations were determined using MEDIPS package [40]. The differentially methylated regions (DMRs) between normal retina and Y79 were detected by filtering the SICER output for peak clusters showing ≥ 2-fold difference in normalized read counts with at least 0.7 reads per million (rpm) and a FDR threshold of 0.01. The DMRs between the control and UHRF1 knockdown Y79 cells were determined by taking the regions overlapping at least in two sets out of triplicate libraries and showing ≥ 1.5-fold difference in normalized read counts with a FDR < 0.05. The annotation of DMRs was carried out by using *annotatePeaks.pl* from Homer toolkit [41]. The analysis of repetitive elements was performed by taking the reads mapped to human hg19 repeat sequences downloaded from UCSC database, and the read counts for each repeat category were normalized by the number of total mapped reads/10^6^ of the corresponding libraries. MeDIP-seq data in this study were deposited in the NCBI Gene Expression Omnibus (GEO) database under the accession number GSE92712.

### Statistical analyses

Statistical significance was determined from at least three independent experiments by two-tailed unpaired student's t-test using GraphPad Prism unless indicated otherwise in the legend.

## SUPPLEMENTARY FIGURES AND TABLES









## Abbreviations

NR: normal retina
5meC: 5-methylcytosine
MSP: methylation-specific PCR
MeDIP: methylated DNA-immunoprecipitation
DMR: differentially methylated region
