# Supplementary material for: Functional dissection of the role of UHRF1 in the regulation of retinoblastoma methylome
Source: Oncotarget. 2017 Apr 13;8(24):39497–511. doi: 10.18632/oncotarget.17078 (PMC5503627; doi:10.18632/oncotarget.17078)
Supplement: Supplementary file 1 [file oncotarget-08-39497-s001.pdf]

Functional dissection of the role of UHRF1 in the regulation of retinoblastoma methylome

SUPPLEMENTARY FIGURES AND TABLES

A

	Age (year)
hNR2	42
hNR3	40
hNR4	51
hNR5	36
hNR6	12

B

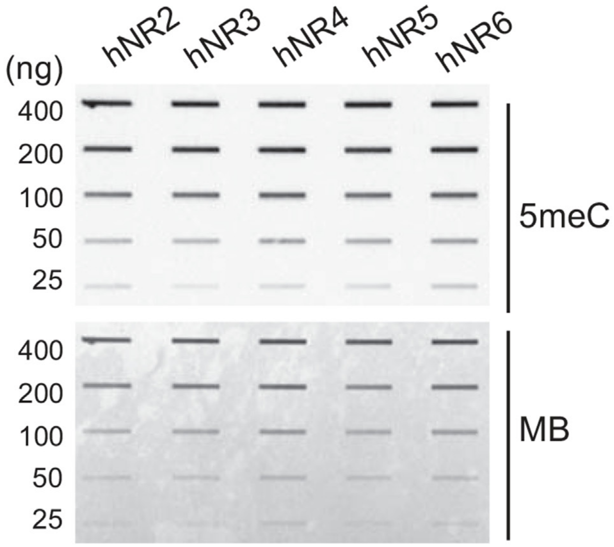

Supplementary Figure 1: Global DNA methylation levels in human normal retina tissues of various age. (A) Table showing the age of normal retina (hNR) tissue donors at the time of tissue collection. **(B)** Total 5-methylcytosine (5meC) level in hNR tissues described in **(A)** with methylene blue (MB) staining of the membrane as a loading control.

shUHRF1-1: AAGAAGGAACGAATCAAAGGC

shUHRF1-2: AAAGCAGTTGAGAGCCAGCGC

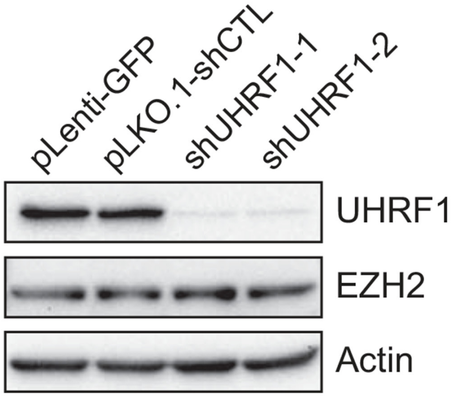

Supplementary Figure 2: Knockdown efficiency of two lentiviral shRNA constructs for UHRF1 in Y79 cells in comparison with lentiviral GFP and control shRNA constructs. Mature antisense sequences of the two UHRF1 shRNA constructs are shown, and EZH2 immunoblot demonstrates that the two UHRF1 shRNA constructs are specific for UHRF1.

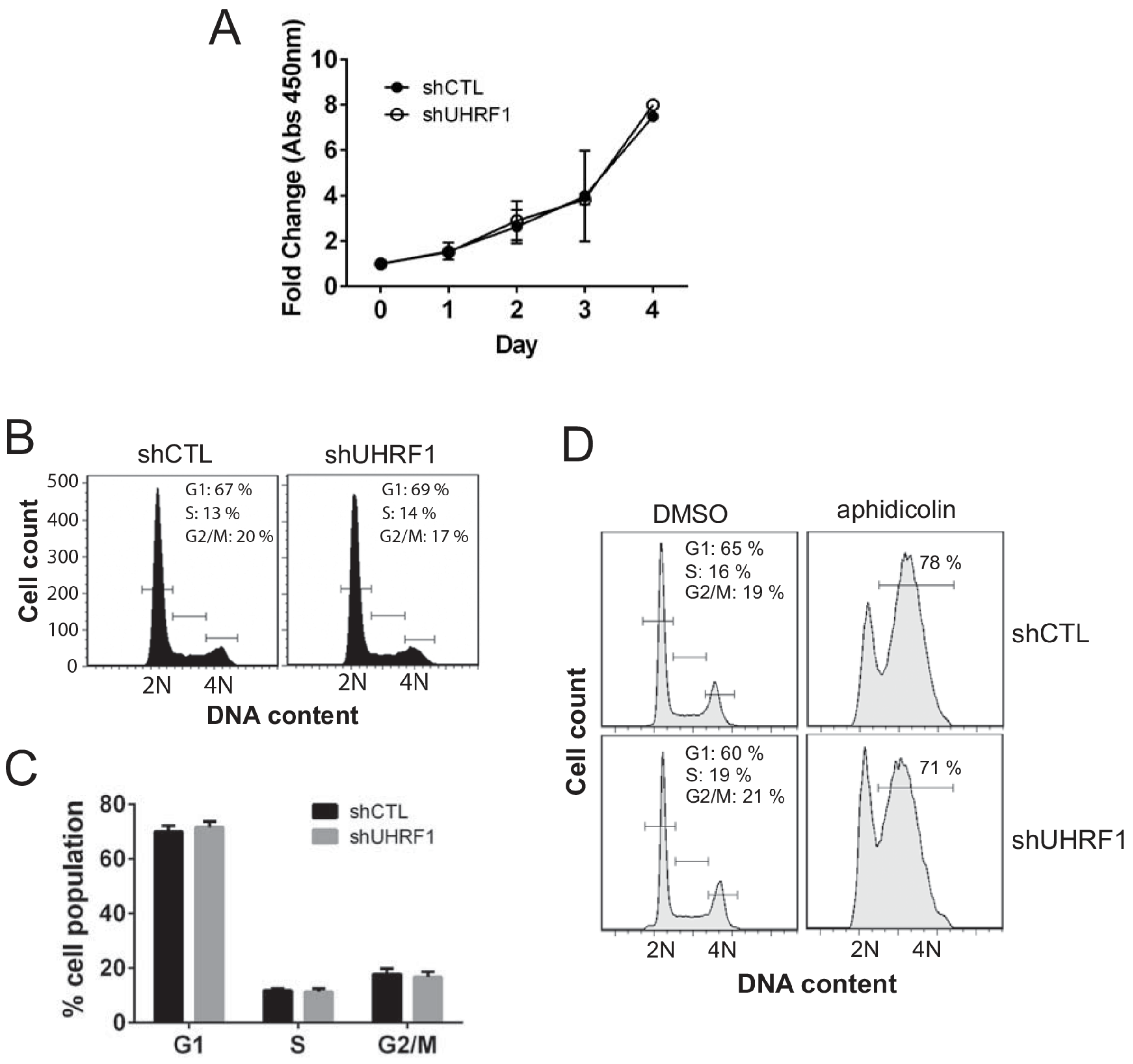

Supplementary Figure 3: UHRF1 depletion does not affect cell proliferation and cell cycle profiles in Y79 cells. (A) Cell proliferation curve determined by Cell Counting Kit-8 (CCK8) assay, using stable UHRF1 knockdown Y79 cells. Absorbance values at each day were divided by those at Day 0 for fold changes in proliferation. The data are shown as the mean ± standard deviation (SD) of fold changes from four independent experiments. **(B)** Representative cell cycle profiles of control and UHRF1 knockdown Y79 cells. **(C)** Graph showing the % cell population at each cell cycle phase upon stable UHRF1 knockdown in Y79 cells. The graph is shown as the mean ± SD from five independent experiments. **(D)** Both control and UHRF1 knockdown Y79 cells progress through S phase in a comparable manner upon release from the aphidicolin block (early S phase arrest). The cell cycle profiles at 6 hr release from DMSO or aphidicolin treatment for indicated cells are shown.

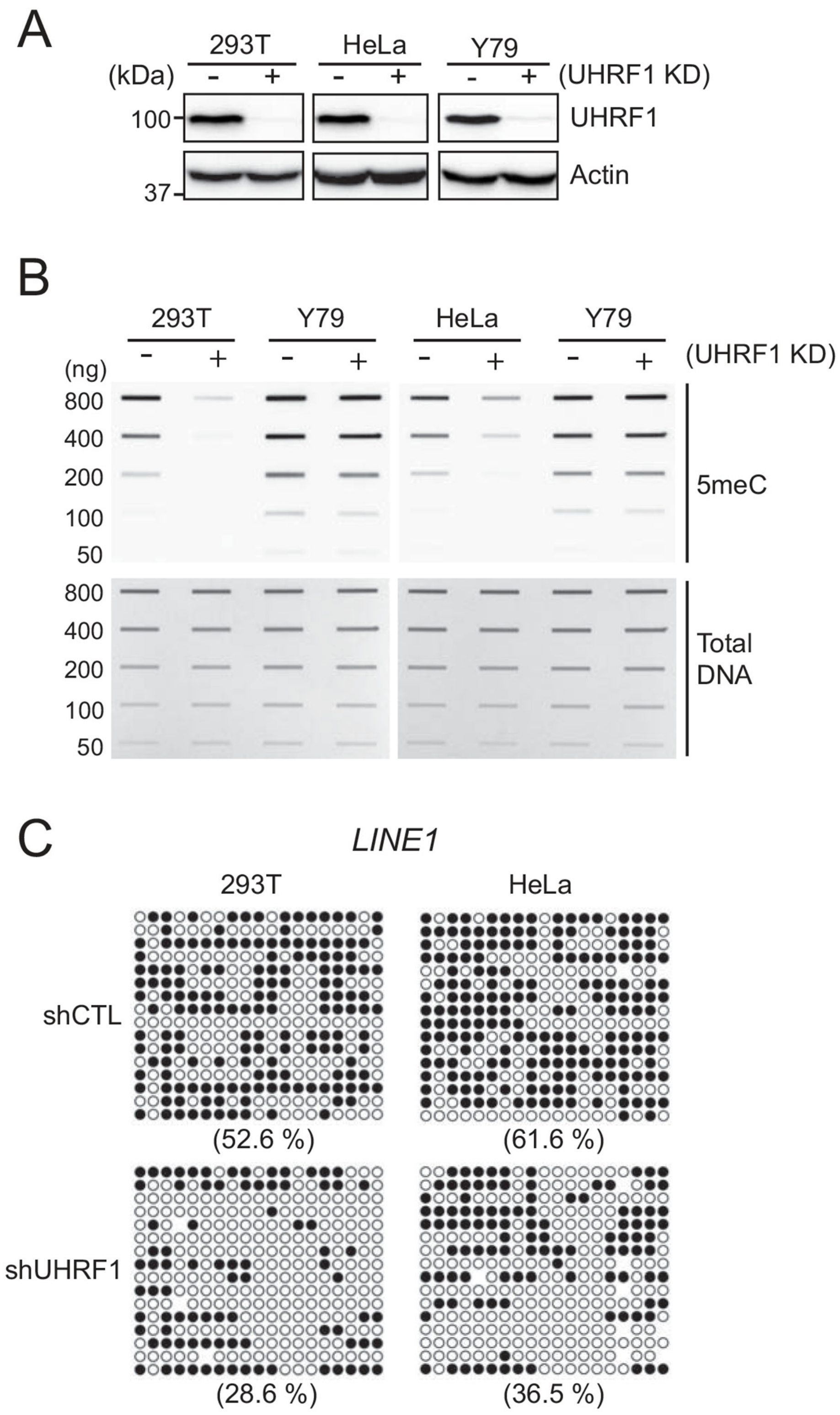

Supplementary Figure 4: UHRF1 depletion results in global hypomethylation in 293T and HeLa cells. **(A)** UHRF1 depletion in three cell lines indicated, following the identical lentiviral shRNA-mediated stable knockdown. **(B)** Slot blot analyses for the total 5meC level in 293T and HeLa in parallel with Y79 after stable knockdown of UHRF1. **(C)** Bisulfite sequencing showing methylation patterns at LINE1 in stable UHRF1 knockdown 293T and HeLa cells. Percentage of methylated CpG dinucleotides is shown in parentheses.

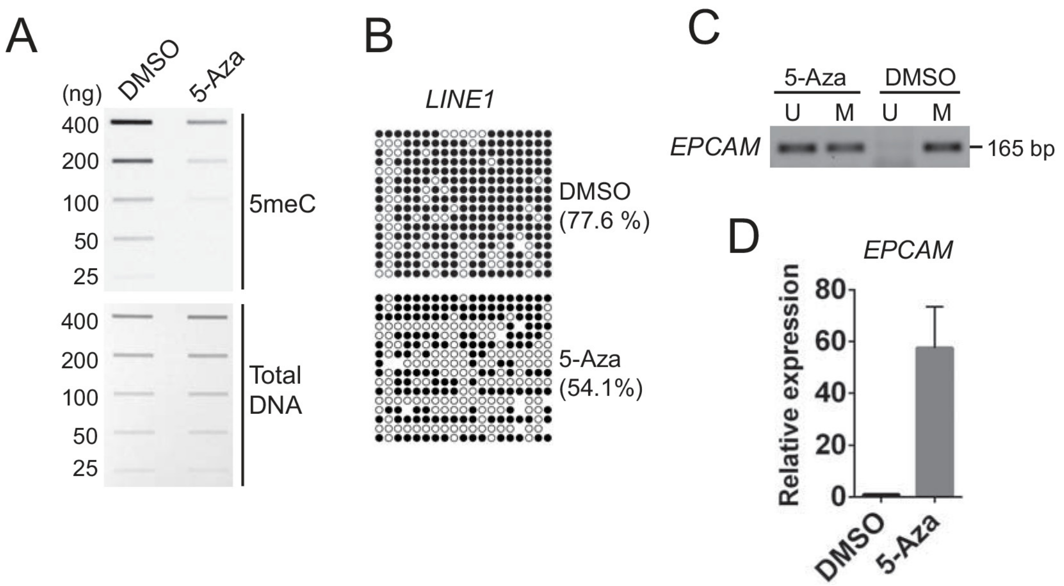

Supplementary Figure 5: 5-Azacytidine treatment induces global hypomethylation and re-expression of tumor suppressor EPCAM in Y79 cells. Y79 cells were treated with DMSO or 1 μM 5-Azacytidine (5-Aza) for 5 days by adding the drug freshly every day. **(A)** Slot blot analysis for the total 5meC in cells indicated. **(B)** Bisulfite sequencing for LINE1 showing the percentage of methylated CpG dinucleotides in parentheses. **(C)** MSP for *EPCAM* promoter showing significant demethylation upon 5-Aza treatment. The PCR products of unmethylated (U) and methylated (M) primers are shown. **(D)** Re-expression of EPCAM following 5-Aza treatment, determined by qRT-PCR analyses. The graph is shown as the mean ± SD of fold changes from two independent experiments, relative to the normalized EPCAM expression level in DMSO-treated Y79 cells.

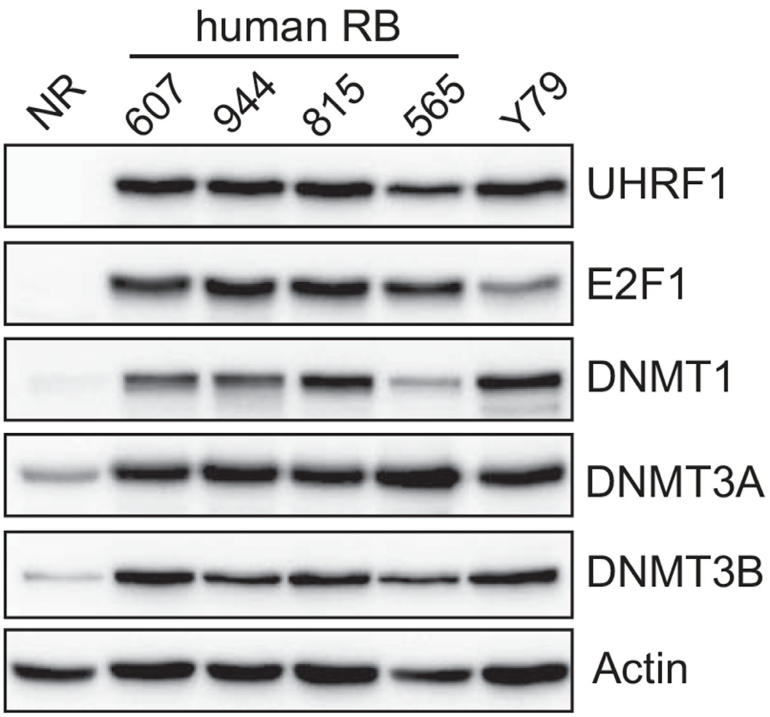

Supplementary Figure 6: Expression of DNMT family proteins in human primary retinoblastoma and Y79 cell line. Western blot analyses for UHRF1, E2F1, and DNMT family proteins in primary retinoblastoma and Y79 cells are shown in comparison with normal retina (NR, 12 years).

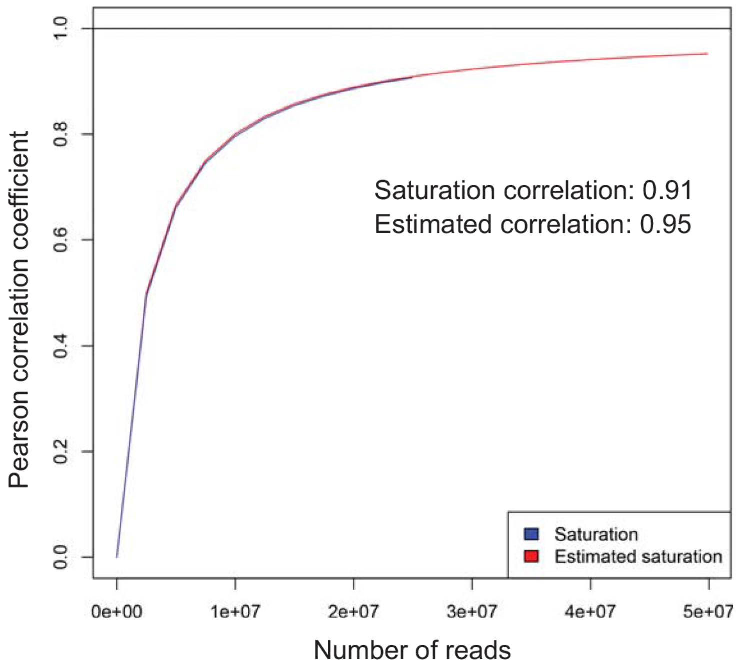

Supplementary Figure 7: Saturation analysis with uniquely mapped reads. One set of the PE125 sequencing data in this study was taken as an example to assess whether the number of reads is sufficient to result in a saturated coverage profile of the reference genome by MEDIPS package. Blue line represents the saturation profile generated by the actual sequencing data while red line indicates the estimated saturation curve. Pearson correlation coefficients of the two saturation curves are shown.

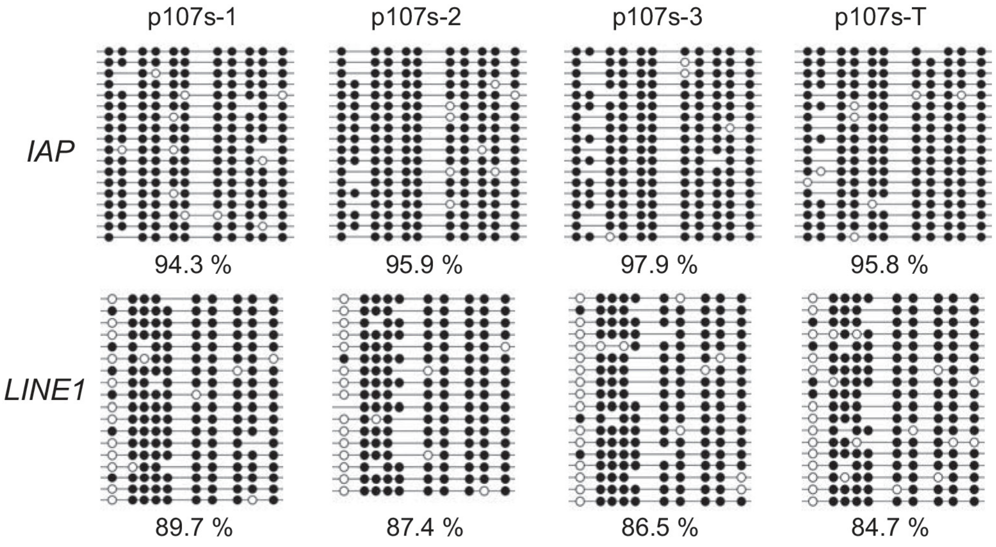

Supplementary Figure 8: Methylation at IAP and LINE1 in p107s. Bisulfite sequencing results are shown for the p107s P8 retinae (1-3) and tumor (T) described in Fig. 6C. Methylated and unmethylated CpG dinucleotides are represented by closed and open circles, respectively. Percentage of methylated CpG dinucleotides is shown below.

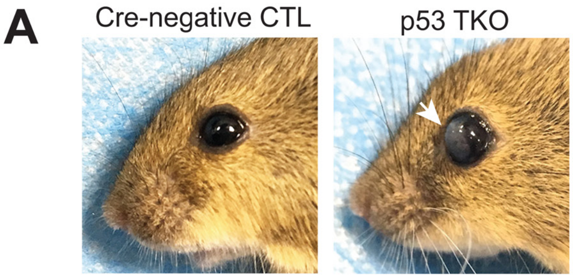

B

blot 1 mouse ID	blot 1 tumor onset (month)	blot 2 mouse ID	blot 2 tumor onset (month)	blot 3 mouse ID	blot 3 tumor onset (month)
246	2.5	841	2.6	991	2.8
732	2.3	922	2.7	988	2.8
814	2.6	995	2.4	708	6.5
145	5.4	394	6.8		
360	6.1	366	9.4		

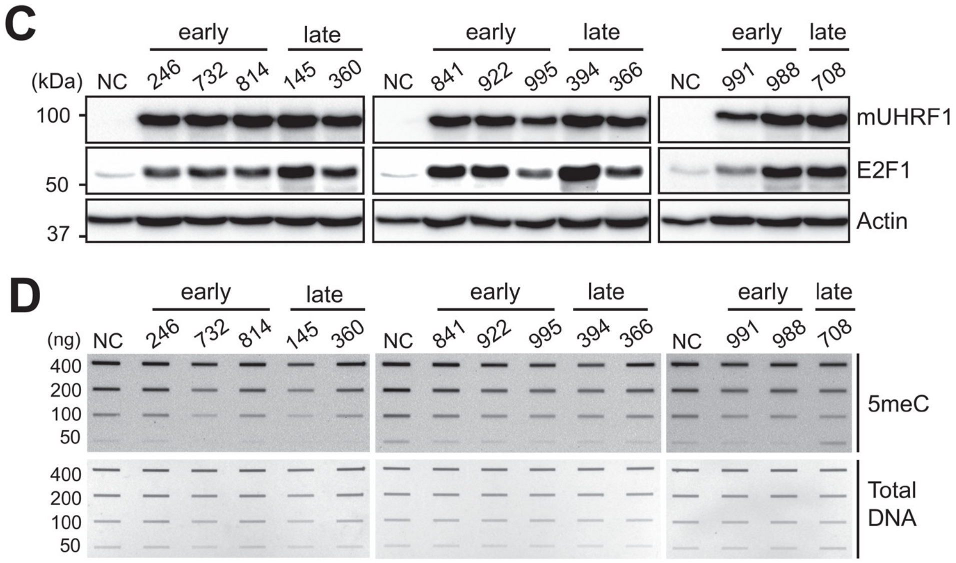

Supplementary Figure 9: Global hypomethylation may not be an early event leading to retinoblastoma development. **(A)** Representative images of Cre-negative control and tumor-burdened eyes of the p53 TKO mice used in this study. A white arrow indicates anterior chamber invasion of tumor cells in the eye. **(B)** Tables of p53 TKO mice with a different tumor onset time. **(C)** Expression of mouse UHRF1 (mUHRF1) and E2F1 in the corresponding tissues shown in **(B)**. **(D)** Slot blot analyses for the total 5meC level in the corresponding tissues shown in **(B)**.

Primers used in this study

Quantitative RT-PCR	
(1) UHRF1	Product size
Forward: 5'-CAAGATTGAGCGGCCGGGTGAAGG-3'	
Reverse: 5'-TGAGGGGCGGGTCCAGGCAGTAGA-3'	214 bp
(2) RARβ	
Forward: 5'-GGGTCAATCCACTGAAGCAT-3'	
Reverse: 5'-GACTGTATGGATGTTCTGTC-3'	118 bp
(3) CDH1	
Forward: 5'-CCCGCCTTATGATTCTCTGCTCGTG-3'	
Reverse: 5'-TCCGTACATGTCAGCCAGCTTCTTG-3'	160 bp
(4) EPCAM	
Forward: 5'-CGCAGCTCAGGAAGAATGTG-3'	
Reverse: 5'-TGAAGTACACTGGCATTGACG-3'	98 bp
(5) Actin	
Forward: 5'-AGAGCTACGAGCTGCCTGAC-3'	
Reverse: 5'-AGCACTGTGTTGGCGTAC-3'	184 bp
**Methylation-specific PCR (MSP)**	
(1) RARβ2	
UM-Forward: 5'-TTGAGAATGTGAGTGATTTGA-3'	
UM-Reverse: 5'-AACCAATCCAACCAAAACAA-3'	146 bp
M-Forward: 5'-TCGAGAACGCGAGCGATTCG-3'	
M-Reverse: 5'-GACCAATCCAACCGAAACGA-3'	146 bp
(2) CDH1	
UM-Forward: 5'-TAATTTTAGGTTAGAGGGTTATTGT-3'	
UM-Reverse: 5'-CACAACCAATCAACAACACA-3'	97 bp
M-Forward: 5'-TTAGGTTAGAGGGTTATCGCGT-3'	
M-Reverse: 5'-TAACTAAAAATTCACCTACCGAC-3'	116 bp
(3) EPCAM	
UM-Forward: 5'-TTTAATGTTGTTATGGAGATGA-3'	
UM-Reverse: 5'-ACCACTAATACTCATTAATAAATCACCAC-3'	165 bp
M-Forward: 5'-TTTAACGTCGTTATGGAGACGA-3'	
M-Reverse: 5'-GCTAATACTCGTTAATAAATCACCG-3'	165 bp
**Bisulfite sequencing**	
(1) Human LINE1	
Forward: 5'-TAGGGAGTGTTAGATAGTGG-3'	
Reverse: 5'-CCCTCTAAACCAAATATAAAATATAATC-3'	242 bp
(2) Mouse LINE1	
Outer forward: 5'-GTTAGAGAATTTGATAGTTTTTGGAATAGG-3'	
Outer reverse: 5'-CCAAAACAAAACCTTTCTCAAACACTATAT-3'	
Inner forward: 5'-TAGGAAATTAGTTTGAATAGGTGAGAGGT-3'	
Inner reverse: 5'-TCAAACACTATATTACTTTAACAATTCCCA-3'	281 bp
(3) Mouse IAP	
Outer forward: 5'-TTGATAGTTGTGTTTTAAGTGGTAAATAAA-3'	
Outer reverse: 5'-AAAACACCACAAACCAAAATCTTCTAC-3'	
Inner forward: 5'-TTGTGTTTTAAGTGGTAAATAAATAATTTG-3'	
Inner reverse: 5'-CAAAAAAAACACACAAACCAAAAT-3'	262 bp
**MeDIP-qPCR**	
(1) CDH1	
Forward: 5'-TAGAGGGTCACCGCGTCT-3'	
Reverse: 5'-CGGCCACAGCCAATCAGC-3'	90 bp
(2) EPCAM	
Forward: 5'-GCCTGTGTTTGTATTTCCTTTAG-3'	
Reverse: 5'-CTGTGCTGAGACTTCCTTTTAAC-3'	125 bp
(3) RASSF1	
Forward: 5'-CCGACCTATCTCAGTGGGTTAC-3'	
Reverse: 5'-CTAGGCGATAGAGATCCAAC-3'	129 bp
(4) DDX1	
Forward: 5'-GGCAGGCTACAGATAGCACA-3'	
Reverse:5'-AGATGAGGACTCTAAAATTCGG-3'	130 bp
(5) LRCH1	
Forward: 5’-CACTTGCAAGCGGACATGTTC-3’	
Reverse: 5’-AAACAGCAAATCAGTCACAGCTC-3’	113 bp
(6) ABHD6	
Forward: 5’-AACGACCATAGCAGGGGTTTAAC-3’	
Reverse: 5’-TGTGCGGGACGTCAGACT-3’	135 bp

Supplementary Table 1: Sequence read generation and alignment

(1) Normal retina vs. Y79 (PE125)

Sample name	Total number of reads	Uniquely mapped reads*	% Uniquely mapped reads	CpG enrichment (relative frequency)#
MeDIP_ NR	286,55,493	219,23,984	76.5%	1.94
MeDIP_Y79	322,07,663	216,25,637	67.1%	3.14
Input_ NR	350,77,681	285,76,521	81.5%	1.05
Input_ Y79	444,54,398	358,27,497	80.6%	1.05
**Sum**	1403,95,235	1079,53,639	NA	NA
**Average per sample**	350,98,809	269,88,410		

(2) Y79 shCTL vs. Y79 shUHRF1 (SE50)

Sample name	Total number of reads	Uniquely mapped reads*	% Uniquely mapped reads	CpG enrichment (relative frequency)#
MeDIP_ shCTL-1	503,99,348	261,03,718	51.8%	2.69
MeDIP_ shCTL-2	575,76,887	300,35,706	52.2%	2.65
MeDIP_ shCTL-3	512,22,877	291,46,953	56.9%	2.99
MeDIP_ shUHRF1-1	413,44,037	209,80,630	50.7%	2.76
MeDIP_ shUHRF1-2	383,24,748	196,41,075	51.2%	2.72
MeDIP_ shUHRF1-3	687,83,848	360,80,215	52.5%	2.87
IgG_shCTL	426,11,621	262,39,642	61.6%	1.09
IgG_shUHRF1	556,32,967	360,80,215	64.9%	1.04
**Sum**	4058,96,333	2243,08,154	NA	NA
**Average per sample**	507,37,042	280,38,519		

*: Uniquely mapped reads exclude duplicates and repetitive genomic regions.
#: Relative frequency of CpG enrichment calculated by the MEDIPS package.

Supplementary Table 2: List of DMR promoters in Y79 cells in comparison with normal retina

Due to the size limit, only promoter DMRs are listed in the table. The complete DMR list can be provided upon request

See Supplementary File 1

Supplementary Table 3: List of DMRs in UHRF1 knockdown Y79 cells compared with control knockdown counterpart

See Supplementary File 2

Supplementary Table 4: Pearson's correlation between MeDIP-seq datasets

See Supplementary File 3
